# Speciation of iron(II/III) at the iron-cement interface: a review

**DOI:** 10.1617/s11527-023-02115-x

**Published:** 2023-02-08

**Authors:** Erich Wieland, George Dan Miron, Bin Ma, Guoqing Geng, Barbara Lothenbach

**Affiliations:** 1 Laboratory for Waste Management, Paul Scherrer Institut, Villigen PSI, Switzerland; 2 Department of Civil and Environmental Engineering, National University of Singapore, Singapore, 117576 Singapore; 3 Concrete & Asphalt Laboratory, Empa, Dübendorf, Switzerland; 4 Institute of Geological Sciences, University of Bern, Bern, Switzerland

**Keywords:** Ferrous iron, Ferric iron, Steel, Cement, Thermodynamic modelling

## Abstract

Steel is used as reinforcement in construction materials and it is also an important component of cement-stabilized waste materials to be disposed of in deep geological repositories for radioactive waste. Steel corrosion releases dissolved Fe(II/III) species that can form corrosion products on the steel surface or interact with cementitious materials at the iron-cement interface. The thermodynamically stable Fe species in the given conditions may diffuse further into the adjacent, porous cement matrix and react with individual cement phases. Thus, the retention of Fe(II/III) by the hydrate assemblage of cement paste is an important process affecting the diffusive transport of the aqueous species into the cementitious materials. The diffusion of aqueous Fe(II/III) species from the steel surface into the adjacent cementitious material coupled with the kinetically controlled formation of iron corrosion products, such as by Fe(II) oxidation, decisively determines the extension of the corrosion front. This review summarises the state-of-the art knowledge on the interaction of ferrous and ferric iron with cement phases based on a literature survey and provides new insights and proper perspectives for future study on interaction systems of iron and cement.

## Introduction

**Fig. 1:**
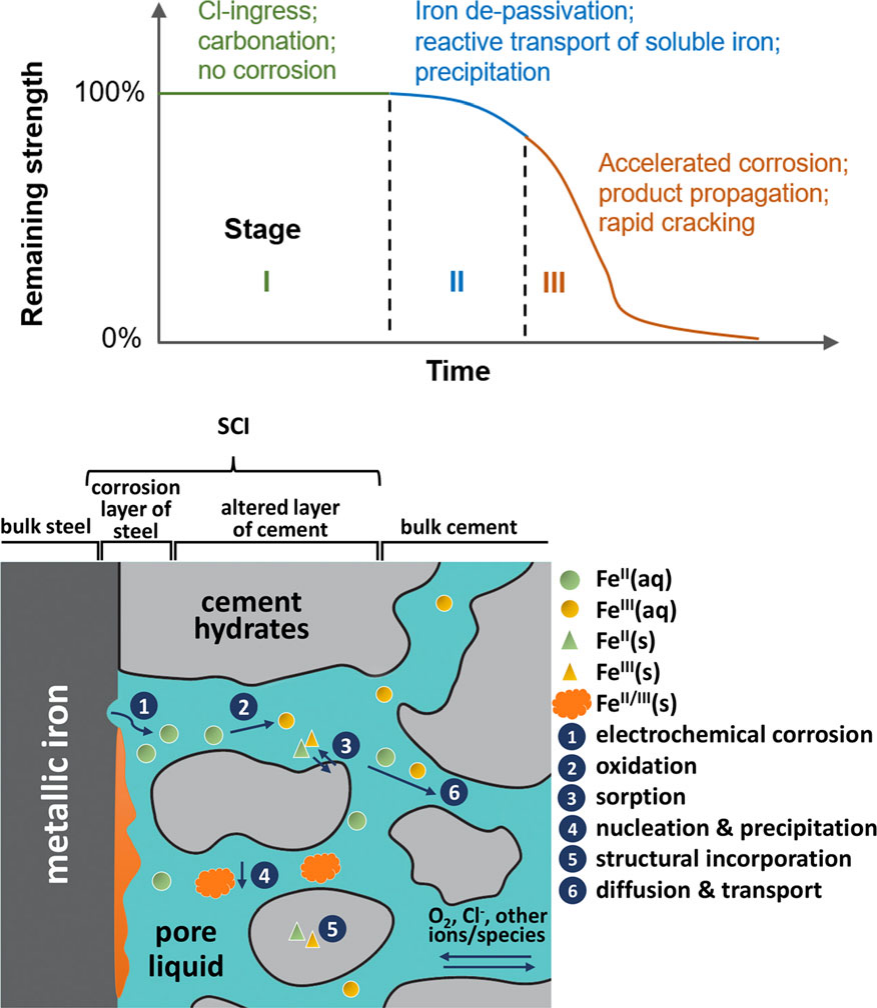
Top: Simplified three-stage corrosion deterioration in reinforced concrete (curve not to scale), adapted from [15]. Bottom: Schematic illustration of the steel–concrete interface (SCI) and of the interrelated processes (labelled with numbers 1–6) governing the corrosion (adapted from Angst et al. [2])

De-passivation and corrosion of steel bars in concrete can lead to considerable damage on a structural level. Reinforcement corrosion can impair the structural serviceability and load bearing capacity because of cracking and spalling of the concrete cover, loss of bonds at the steel–concrete interface and reduction of the sectional area of reinforcing steel as summarised in recent reviews [1, 2]. The Fe(II) and Fe(III) generated by the corrosion of steel are expected to affect also the mineral composition of hydrated cement in the long term. The changes in the hydrates will occur at the iron/steel–concrete interface or near to it as reported by Angst et al. [2] and illustrated in Fig. 1.

In addition to reinforcing steel in concrete structures, metallic iron in cement can originate from ground granulated blast furnace slag (GGBFS) or non-ferrous metallurgy slag, which can contain a substantial amount of Fe(0) present as finely dispersed metallic nano- to micron-sized particles [3–5]. To date, limited information is available on the long-term fate of this nano- to micron-sized Fe(0) in alkali-activated slags or in blends with Portland cement (PC). It is conceivable that the corrosion behaviour of Fe(0) in slag cements is similar to that of reinforcing iron (and steel) bars in concrete structures, although the kinetics might be different due to the large surface area of the micron-sized granules as compared to reinforcing bars and as GGBFS can lead to reducing conditions in the pore solution [6, 7]. Studies on the corrosion of reinforcing bars in concrete show that the corrosion rate is controlled by the multiple factors, including the chemical conditions (i.e. pH, redox conditions, chloride and other elemental content of concrete pore solution, etc.), the metallography of the rebar, the pore structure of concrete and the moisture condition near the interface [8–10].

It is agreed that the corrosion of reinforced concrete takes place in three kinetic stages (Fig. 1). Chloride ingress and carbonation reaching the steel–concrete interface often mark the end of stage I, during which the steel is not corroded. In stage II, the oxidation of iron is activated, and the dissolved iron species undergo a reactive transport in the cement paste, precipitating in the pore space and/or taken up by the hydration products. When the expansive corrosion product generates sufficient micro-cracks, the concrete cover becomes much more pervious to chloride, water and oxygen, driving the degradation to stage III when the structure quickly loses its strength. The modelling of stage I has been widely reported and validated [11–14], whereas it is still very challenging to model stage II. This is largely due to the limited understanding of the initial corrosion product formation and interaction with porous cement paste. The unpredicted duration of stage II limits the decision of the best maintenance time, as it is too early to take any action in stage I, but too late in stage III.

The corrosion of iron and steel releases ferrous Fe(II) and ferric Fe(III) species, which either form corrosion products on the steel surface or diffuse into the adjacent cementitious material, thus giving rise to interaction with cement phases or, depending on solubility, to the formation of specific Fe-containing minerals [16]. Reactive transport models have been developed that allow steel corrosion and the distribution of corrosion products to be predicted in concrete structures [17, 18] and in connection with the long-term safe disposal of radioactive waste in deep geological repositories (DGR) [19–26]. Depending on the application, the models have been based on a series of parallel processes mimicking steel dissolution and interaction with adjacent materials. For example, iron dissolution, diffusion and oxidation of ferrous iron as well as precipitation of ferrous and ferric iron-containing minerals have been considered for applications in concrete structures [16–18].

The diffusion of the aqueous species is an important transport mechanism in the porous media, which is driven by the gradient in the concentration of the solutes. The molar flux due to molecular diffusion in the porous media is characterized by an apparent diffusion coefficient, which depends on the diffusion constant, the accessible porosity of the material, the geometry of the pore network (constrictivity, tortuosity) and, in the case of sorbing ions, on the sorption properties of the ion [12, 27]. In cementitious environments, the mobility and thus the diffusion distance depend decisively on the interaction of the diffusing ion with individual cement minerals or the formation of a precipitate accommodating the ion.

Depending on the physico-chemical conditions (solution properties, availability of moisture, temperature, etc.), different types of Fe-containing cement phases and corrosion products are stable. These exhibit a wide range of properties, e.g. ranging from highly soluble to insoluble solids, and variable volumes that affect the porosity and permeability, material performance and the retention and transport of iron from the steel–concrete interface. The evolution of important chemical properties, such as redox, pH, ionic strength etc., and solid-phase parameters, such as mineralogy, porosity etc., at the steel–concrete interface is governed by the Fe-containing phases that are formed and their stability under the evolving conditions [21]. Knowledge of the interaction of ferrous and ferric iron species with cement phases and thermodynamic data on the stability of the iron-containing phases as well as on the kinetics of phase formation are therefore required to accurately model the long-term behaviour of iron/steel at the interface with cementitious materials.

The aim of this review paper is to discuss the chemical processes involving ferrous and ferric iron at the iron/steel–concrete interface based on new literature with a view to possible impacts on the long-term stability of cementitious materials. To this end, state-of-the art knowledge on the nature of the iron corrosion products and the interaction of Fe(II) and Fe(III) with cement phases is summarised and a preliminary assessment of the long-term evolution of the iron-cement interface is undertaken.

## Corrosion products on the steel surface

In cementitious environment, metallic iron is thermodynamically not stable and subject to corrosion. A dense oxide film on the iron/steel surface may inhibit further corrosion, which however is readily destabilized by Cl^−^ ions and/or decreased pH [1]. The active iron in aqueous media is described in terms of an electrochemical process involving two separate, but coupled anodic and cathodic processes proceeding simultaneously [28]. The anodic process involves the oxidation of iron, which produces ferrous (Fe^2+^) or ferric (Fe^3+^) ions and releases electrons. The electrons are subsequently consumed by a cathodic process, which involves the reduction of an oxidising agent acting as electron acceptor. Overall, electrical neutrality is preserved.

In most engineered structures, including in the early phase of a DGR, the corrosion of iron/steel under aerobic conditions will initially proceed by the reduction of oxygen (electron acceptor). Under these conditions, the common corrosion products identified are Fe(III) (oxy)hydroxides and oxides. In the long term in DGRs [29–31] and near the steel surface, the redox conditions can evolve from oxidising to reducing conditions due to consumption of the available oxygen and reduction of the oxygen fugacity. In the absence of oxygen or other oxidising agents, iron corrosion involves the reduction of water, which results in the production of hydrogen and the formation of Fe(II/III) corrosion products, in particular magnetite.

The main products formed during corrosion under anaerobic conditions are magnetite (Fe_3_O_4_) [32, 33] and “black rust”, which is mainly composed of magnetite as well [28, 34]. The aerobic corrosion of iron and steel gives rise to the formation of Fe(III) oxyhydroxides and oxides, e.g. ferrihydrite (FeOOH·xH_2_O), lepidocrocite (γ-FeOOH) and goethite (α-FeOOH) in concrete structures. These minerals can further crystallise to maghemite (γ-Fe_2_O_3_) and hematite (α-Fe_2_O_3_)), while magnetite (Fe(II)Fe(III)O_4_) may also be present [28, 35–37]. Corrosions products have further been characterised in archaeological samples that had undergone corrosion under aerobic conditions in a natural environment over a very long period of time [38, 39]. Under these conditions, goethite (α-FeOOH) and magnetite/maghemite (Fe_3_O_4_/γ-Fe_2_O_3_) were identified as the main corrosion products using classical laboratory techniques, such as X-ray diffraction (XRD) and Raman spectroscopy [40–43], and modern synchrotron-based micro-spectroscopic techniques [44–46] and micro X-ray diffractional methods [5, 47].

In addition to the main corrosion products, small proportions of siderite (FeCO_3_) and/or basic Fe hydroxychlorides (Fe_2_(OH)_3_Cl, β-FeO_1−*x*_(OH)_1+*x*_Cl_*x*_) were observed. This finding is in agreement with earlier laboratory-scale studies on the formation processes of iron oxyhydroxides during the aerobic corrosion of iron [48–50] and measurements on steel bars embedded in cementitious system over more than 60 years [51].

In chloride-bearing environments ferric akaganéite (β-FeO(OH,Cl)) and ferrous hibbingite (γ-Fe_2_(OH)_3_Cl) were identified [52–54]. Fe(OH)_2_ is favoured with increasing pH, while the hydroxychloride phases form in neutral to slightly alkaline environments [53, 55]. In the presence of oxygen, Fe_2_(OH)_3_Cl is unstable in the long term and transforms into magnetite [52].

Mixed Fe(II/III)-bearing intermediates may form during partial oxidation depending on the specific conditions (solution composition, oxygen fugacity etc.). For example, if the pore water contains chloride, sulphate, and carbonate ions, phases such as “green rusts” (GR) can form [55–58]. GRs are layered double hydroxides (LDH) with mixed Fe(II/III) composition that generally form in anaerobic, neutral to alkaline systems in the presence of anions such as carbonate, sulphate, or chloride [58]. In the presence of oxygen, the ferrous corrosion products will be destabilized and depending on the solution properties, GRs will be metastable intermediate products before being transformed into stable oxide (magnetite) or oxyhydroxide phases (goethite, akagéneite, lepidocrocite) [52, 59]. Thus, the long-term stable corrosion products under aerobic conditions are goethite or magnetite. In the presence of magnesium, also stable Fe(II)- or Fe(III)-containing LDH can form [60–62] as well as LDH with mixed Fe(II/III) replacements, as recently reported in slag cements [63].

Siliceous hydrogarnet, the main Fe-containing phase in hydrated PC paste [64, 65], has also been identified as a product formed at the iron-cement interface during anaerobic iron corrosion [33]. Corrosion of steel forms a passive protecting thin film on ordinary reinforcing steel embedded in an alkaline pore solution [8]. Experimental investigations of the corrosion products formed on archaeological artefacts buried in soil, which corresponds to anaerobic corrosion in near-neutral carbonated media, were performed using both classical laboratory [36, 66–69] and synchrotron-based [70] techniques. The formation of ferrous carbonates, e.g. siderite (FeCO_3_), chukanovite (Fe_2_(OH)_2_CO_3_), and/or magnetite (Fe_3_O_4_) was observed depending on the local pH conditions and carbonate concentrations. Hence, the nature of the corrosion products is also influenced by the presence of carbon and sulphur species in solution, which can lead to the formation of iron carbonates [71] and iron sulphides [72] under anaerobic conditions. Ferrous iron carbonates such as FeCO_3_, Fe_2_(OH)_2_CO_3_ and Fe_6_(OH)_12_CO_3_, or the hydroxysalt chukanovite Fe_2_(OH)_2_CO_3_ have been reported [36, 73]. However, there is evidence to suggest that under anaerobic alkaline (pH > 11) conditions at the iron/steel-cement interface and in presence of moderate carbon and sulphur concentrations, the predominant corrosion product is magnetite [74, 75].

The above compilation of selected studies illustrates that a large range of corrosion products under aerobic and anaerobic conditions has been identified, which vary depending on the chemical environment. Over long time scales of centuries and millennia, the thickness of the corrosion layer may reach a few hundred micrometres to even a few millimetres. Several layers with varying thickness have been identified at the iron/steel interface with the adjacent medium (clay, soil, cement paste, concrete etc., see Fig. 1), such as.

(i) the metallic iron,
(ii) a layer of corrosion products of variable porosity and consisting of various iron (oxyhydr)oxides, carbonates, and possibly sulphides, sulphates or phosphates (internal corrosion products),
(iii) a geochemically altered layer of the adjacent medium consisting of minerals typically observed in this medium, minerals with different degrees of iron incorporation and precipitated corrosion products (external corrosion products), and
(iv) the matrix of the adjacent medium itself [38, 39, 76].

## Interaction of ferrous and ferric iron with cement phases

In DGR applications, the fate of ferrous and ferric iron at the interface between iron/steel and the adjacent medium, such as clay and nuclear waste glasses, has been extensively studied with the aim of assessing the long-term performance of the engineered barrier materials as a result of iron/steel corrosion [77–82]. In contrast, there is little information from in-situ corrosion studies on the Fe(II/III) species interacting with cement phases at the steel–concrete interface [33].

At the steel-clay interface in DGR, the formation of poorly crystallised Fe-silicates was observed, indicating that a reaction between Fe(II) or Fe(III) with constituents of the clay matrix occurred [77, 78]. At the steel-glass interface, the direct contact between iron and magnetite, the main corrosion product under anaerobic conditions, with glass was reported to increase alteration of the nuclear waste glasses with subsequent implications for their long-term durability in a DGR [79–82]. It was found that the solution conditions changed significantly when magnetite was added to the system, leading to the resumption of alteration particularly in surface cracks that provided sites for the alteration. These processes resulted in the equilibrium between solution and the glass matrix being restored [82].

At the iron-cement interface, the formation of successive layers of siliceous hydrogarnet and magnetite was observed [33]. The presence of Fe-containing mineral phases with a composition characteristic of the contact medium forming a dense outer corrosion layer is expected to limit corrosion of carbon steel in anaerobic conditions in clay [83] and cementitious systems [33]. The identification of Fe-containing siliceous hydrogarnet suggests that the precipitation of secondary iron-containing cement phases and the interaction of Fe(II) and Fe(III) with existing cement phases in the cement matrix might be a common phenomenon occurring at the steel-concrete interface. The following section therefore summarises the current state of knowledge on the interaction of Fe(II) and Fe(III) with cement phases.

### Uptake of ferric iron by Al-bearing cement hydrates

The hydrate assemblage of a freshly hydrated PC contains in addition to ~ 40 wt.% calcium silicate hydrate (C–S–H) and 20–25 wt.% portlandite, also 10–15 wt.% ettringite, up to 15 wt.% AFm phases, 5 wt.% Fe/Al siliceous hydrogarnet, and 2–3 wt.% other, hydrotalcite-like LDH [84–86].

Ferric iron (Fe(III)) can potentially substitute structural Al(III) in the Al-bearing hydrates such as ettringite (AFt: Ca_6_(Al,Fe)_2_(SO_4_)_3_(OH)_12_·26H_2_O), AFm phases (e.g. monosulphate (Ca_4_(Al,Fe)_2_(SO_4_)(OH)_12_·6H_2_O, monocarbonate (Ca_4_(Al,Fe)_2_(CO_3_)(OH)_12_·5 or 6H_2_O), hemicarbonate (Ca_4_(Al,Fe)_2_(CO_3_)_0.5_(OH)_13_·5.5H_2_O), Friedel’s salt (Ca_4_(Al,Fe)_2_Cl_2_(OH)_12_·4H_2_O)), in hydrotalcite-like LDH (Mg_3_(Al, Fe)(CO_3_)_0.5_(OH)_8_), and in siliceous hydrogarnet (Ca_3_(Al,Fe)_2_(SiO_4_)_0.84_(OH)_8.64_) [61, 62, 64, 87–92]. The formation of mixed Al–Fe solid solutions has been observed for siliceous hydrogarnet, ettringite, the hydrotalcite-pyroaurite solid solution series, monosulphate, hemicarbonate and Friedel’s salt but not for monocarbonate, due to the structural differences between the triclinic monocarboaluminate Ca_4_Al_2_(CO_3_)(OH)_12_·5H_2_O and the trigonal monocarboferrate Ca_4_Fe_2_(CO_3_)(OH)_12_·6H_2_O [87, 93].

**Fig. 2:**
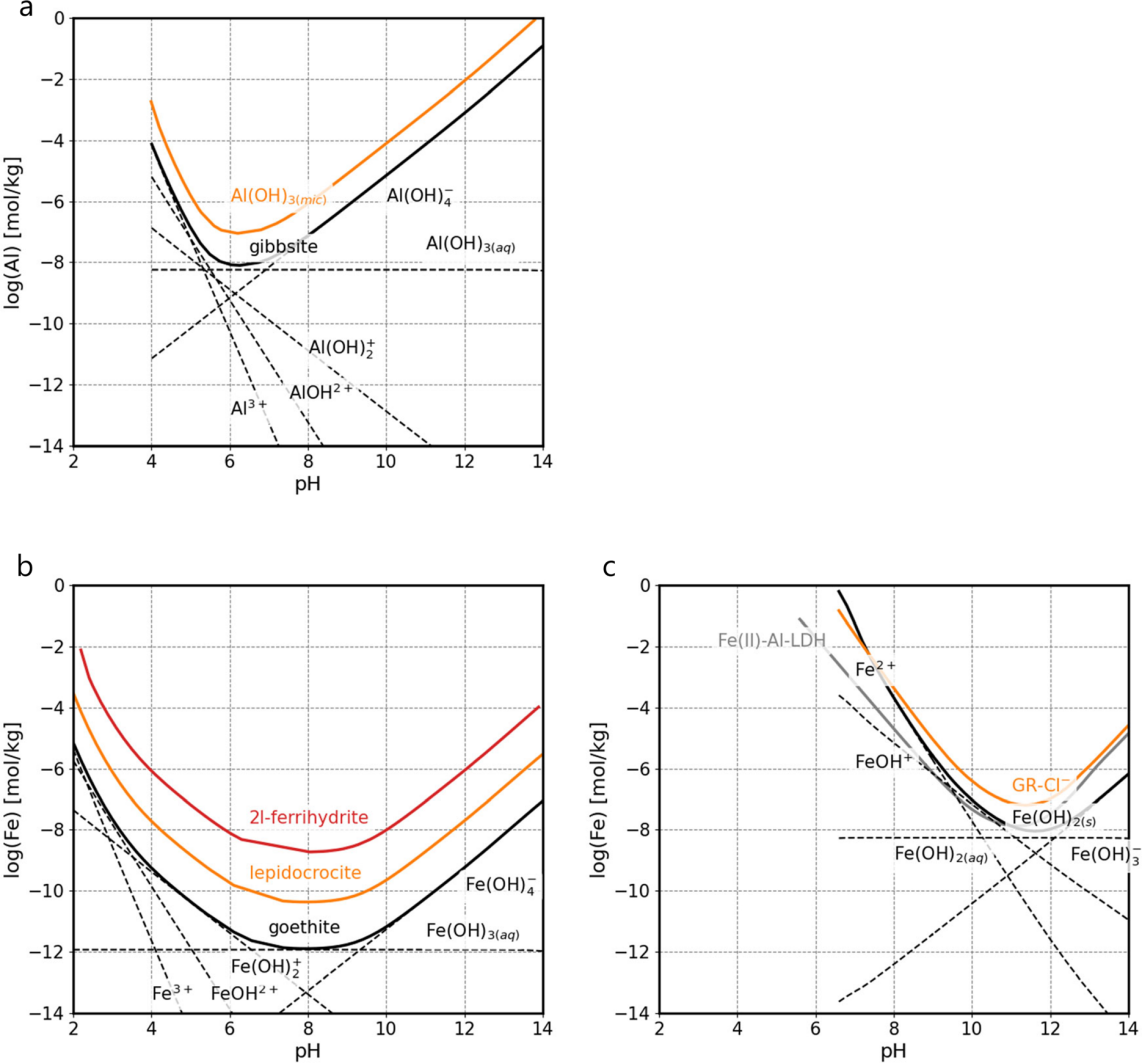
Solubility at 25 °C of **a**) Al (gibbsite (γ-Al(OH)_3_), Al(OH)_3_(mic)), **b**) Fe(III) (goethite (α-FeOOH), lepidocrocite (γ-FeOOH), 2-line-ferrihydrite), and **c**) Fe(II) (Fe(OH)_2_(s)), Green rust-Cl^−^ (Fe(III)_3_Fe(II)(OH)_8_Cl), and Fe(II)-Al-LDH (Fe_2_Al(OH)_6_Cl) calculated using thermodynamic data from Hummel and Thoenen [94] and Bhattacharya and Elzinga [60]

Iron hydroxide generally precipitates initially as an amorphous or microcrystalline phase such as ferrihydrite or lepidocrocite, which recrystallises over time into thermodynamically more stable, crystalline Fe-hydroxide such as goethite (Fig. 2) or hematite. The solubility of iron hydroxide is several log units lower than those of their Al-containing counterparts, i.e. microcrystalline Al(OH)_3_ or gibbsite (γ-Al(OH)_3_) (Fig. 2a, b).

Experimentally derived solubility products of Fe(III)-containing hydrotalcite-like LDH phases are comparable to the corresponding Al-containing phases, while the solubility products of Fe(III)-containing ettringite, monosulphate, monocarbonate, hemicarbonate and Friedel’s salt were found to be approximately 2–3 log units lower than those reported for the Al(III)-containing endmembers [90]. Furthermore, iron hydroxide and siliceous hydrogarnet are considerably more stable than their Al-containing analogues (6 log units lower solubility products) [90]. In fact, Fe/Al siliceous hydrogarnet was identified in cement paste hydrated for 10 and 50 years [65, 95] using XRD, SEM/EDX, and synchrotron-radiation based extended X-ray absorption fine structure (EXAFS) spectroscopy, while ferrihydrite has been identified during the first hours of hydration only [95].

Fe/Al siliceous hydrogarnet was observed in different cementitious environments, such as PC [95, 96] and belite-ye'elimite cements [97, 98]. However, at low belite reaction degree, where the availability of CaO is strongly limited, the possible formation of Fe-containing strätlingite has been reported [97]. The presence of Fe/Al siliceous hydrogarnet was also reported in the degraded surface layer of cement paste leached with demineralised water [99]. Fe(III)-bearing siliceous hydrogarnet was found to readily but slowly form at 20 °C, suggesting significant kinetic control of the formation process, while it was stable up to 110 °C[64]. At ambient temperature, the presence of Fe(III) stabilises Fe/Al siliceous hydrogarnet. Nevertheless, it only forms a poorly crystallised phase in these conditions [90, 95]. Crystalline Fe/Al siliceous hydrogarnet was identified at elevated temperature in fresh cement pastes or in cement pastes that had been aged for several years [65, 95]. Fe/Al siliceous hydrogarnet was also identified as a main product in the corrosion layers of iron in a cementitious environment [33]. This finding supports the idea that the formation of Fe/Al siliceous hydrogarnet is thermodynamically favoured in iron- and calcium-enriched environments.

In cementitious systems with high Mg contents, e.g. due to the addition of granulated blast furnace slag or due to the presence of dolomite, Mg–Al LDHs are formed during hydration [90, 100–102]. The structure of these minerals consists of brucite-like (Mg(OH)_2_) layers, where Mg^2+^ is partially substituted by trivalent cations (Al^3+^, Fe^3+^), thus generating a positive charge of the main layers, which is charge balanced by the uptake of anions in the interlayer (e.g. CO_3_^2−^, SO_4_^2−^, NO_3_^−^, AsO_3_^−^) [103–107]. The interlayer space not occupied by anions is filled with H_2_O. As in the case of AFm phases, Fe^3+^ can replace Al^3+^ in hydrotalcite-like LDH phases [61, 62]. In PC, however, it appears that stabilisation of Fe^3+^ in Fe/Al siliceous hydrogarnet is thermodynamically favoured as compared to the formation of Mg-Fe(III)-LDHs [65, 95], while in low-pH cements rather the formation of ferrihydrite is expected [108].

### Fe(III) bonding to C–S–H phases

Calcium silicate hydrates (C–S–H) are the most abundant cement phase in hydrated PCs and blended cements [109]. C–S–H phases take up Al(III) forming so-called C-A-S–H phases [110–118]. Aluminium is accommodated by the C–S–H phases up to a maximum Al/Si ratio ≈ 0.2 [111–113] and adopts different coordination environments in the C–S–H structure depending on the Ca/Si and Al/Si ratios [113, 119, 120]. ^29^Si nuclear magnetic resonance (NMR) and ^27^Al NMR spectroscopy indicate that Al is mainly coordinated in the bridging position of the silica chains in tetrahedral coordination Al^IV^, although also minor amounts of penta- and octahedrally coordinated Al^V^ and Al^VI^ were observed [113, 114, 117, 120]. XRD further shows that the uptake of Al by C–S–H gives rise to an increase in the interlayer distance [114].

**Fig. 3:**
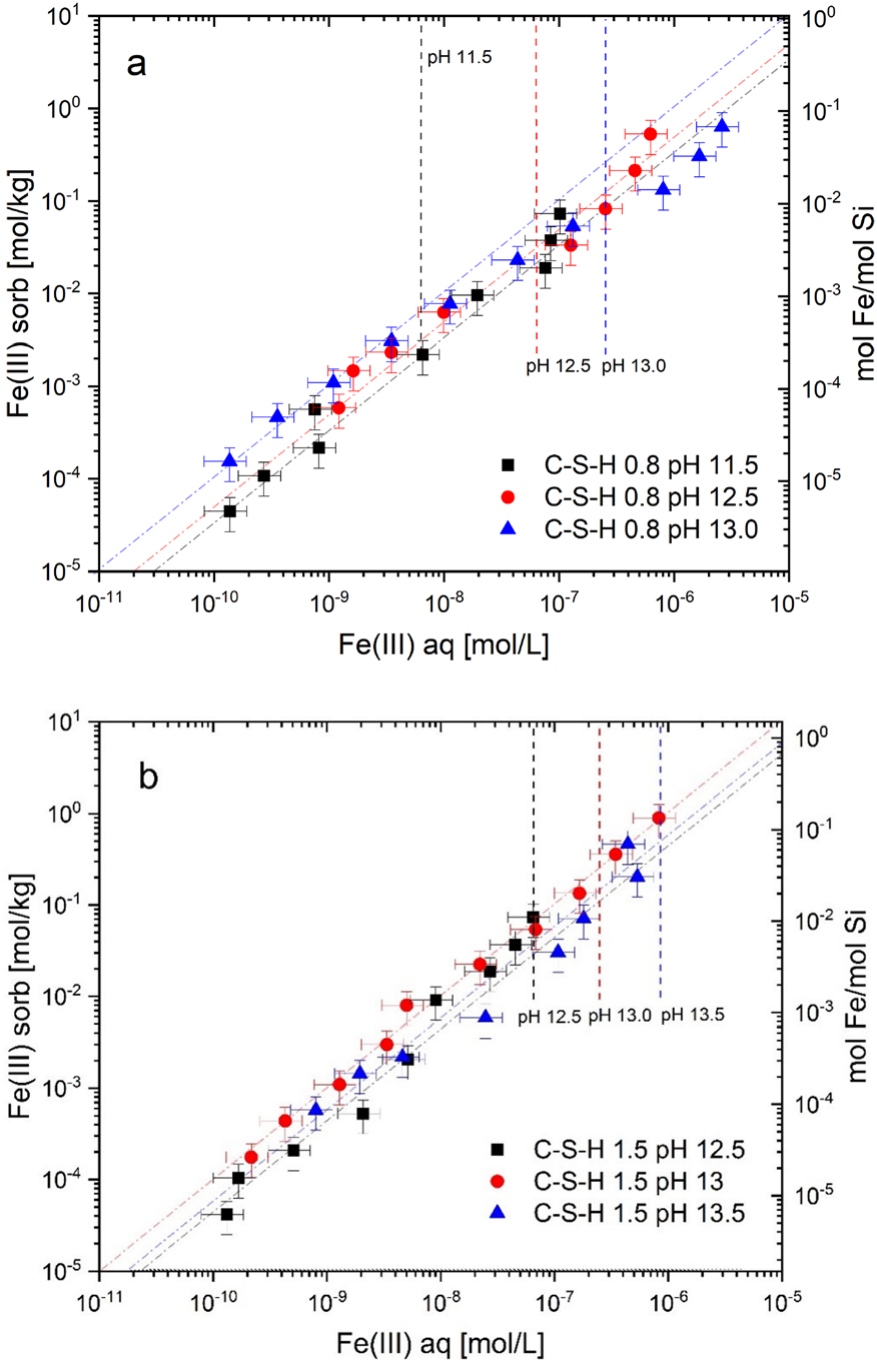
Sorption isotherms of Fe(III) on C–S–H phase with Ca/Si (C/S) ratio = 0.8 (**a**), and C–S–H with C/S ratio = 1.5 (**b**). The experimental data show a linear sorption behaviour (slope = 1 of the dashed lines). Vertical dashed lines show the solubility of lepidocrocite (γ-FeOOH) at given pH values (adapted from Mancini et al. [123])

Similarly to Al(III), also the uptake of Fe(III) by C–S–H has been observed [99, 121–124]. Faucon et al. [99] and Labhasetwar et al. [122] postulated that Fe(III) is taken up via cation exchange with Ca^2+^in the interlayer space of C–S–H phases and further concluded that Fe(III) is present in an octahedral coordination environment in the interlayer based on Mössbauer spectroscopy. More recently, Mancini et al. [123] and Siramanont et al. [124] confirmed that Fe(III) binds strongly to C–S–H phases. Sorption isotherms of Fe(III) determined by Mancini et al. [123] reveal that the sorption behaviour of Fe(III) is linear over the entire aqueous Fe concentration range for C–S–H phases with Ca/Si (C/S) ratios = 0.8, 1.2 and 1.5 (slope = 1, Fig. 3). In contrast to sorption onto TiO_2_, Fe(III) uptake by C–S–H phases was found to linearly increase over the investigated Fe(III) concentration range, implying that ferrihydrite was not a solubility-limiting phase in these systems [123]. Precipitation of amorphous Fe(OH)_3_(am) only occurred at very high Fe concentrations in the C–S–H systems as indicated by EXAFS spectroscopy [123].

The sorption isotherms in Fig. 3 indicate no or only a very weak effect of pH or of the Ca content on Fe(III) uptake by C–S–H phases as the sorption data for Fe(III) uptake by C–S–H phases with target C/S ratios = 0.8 and 1.5 agree within the experimental uncertainty in contrast to Al-uptake by C–S–H (Fig. 5). The pH determines the aqueous speciation of Fe(III); the concentration of Fe(OH)_4_^−^ as well as the total Fe concentration increases with increasing pH above pH 9 (Fig. 2). Due to the increasing Fe(OH)_4_^−^ concentration in the alkaline pH range, the Fe(III) sorption on an inert oxide surface (TiO_2_) was found to decrease with increasing pH in contrast to C–S–H [123].

The iron uptake by C–S–H can also be influenced by the formation of other iron-bearing phases such as siliceous hydrogarnet or ferrihydrite. If other iron-bearing phases stabilise faster, they can outcompete C–S–H for iron binding. The experimental results of Siramanont et al. [124] show that the incorporation of Fe(III) in C–S–H is highly restricted in systems where ferrihydrite initially forms and they suggest that ferrihydrite can form as an intermediate phase before siliceous hydrogarnet formation. In experiments with no precipitation of ferrihydrite, the uptake of Fe(III) in C–S–H was increased, while the formation of siliceous hydrogarnet was slowed down.

**Fig. 4:**
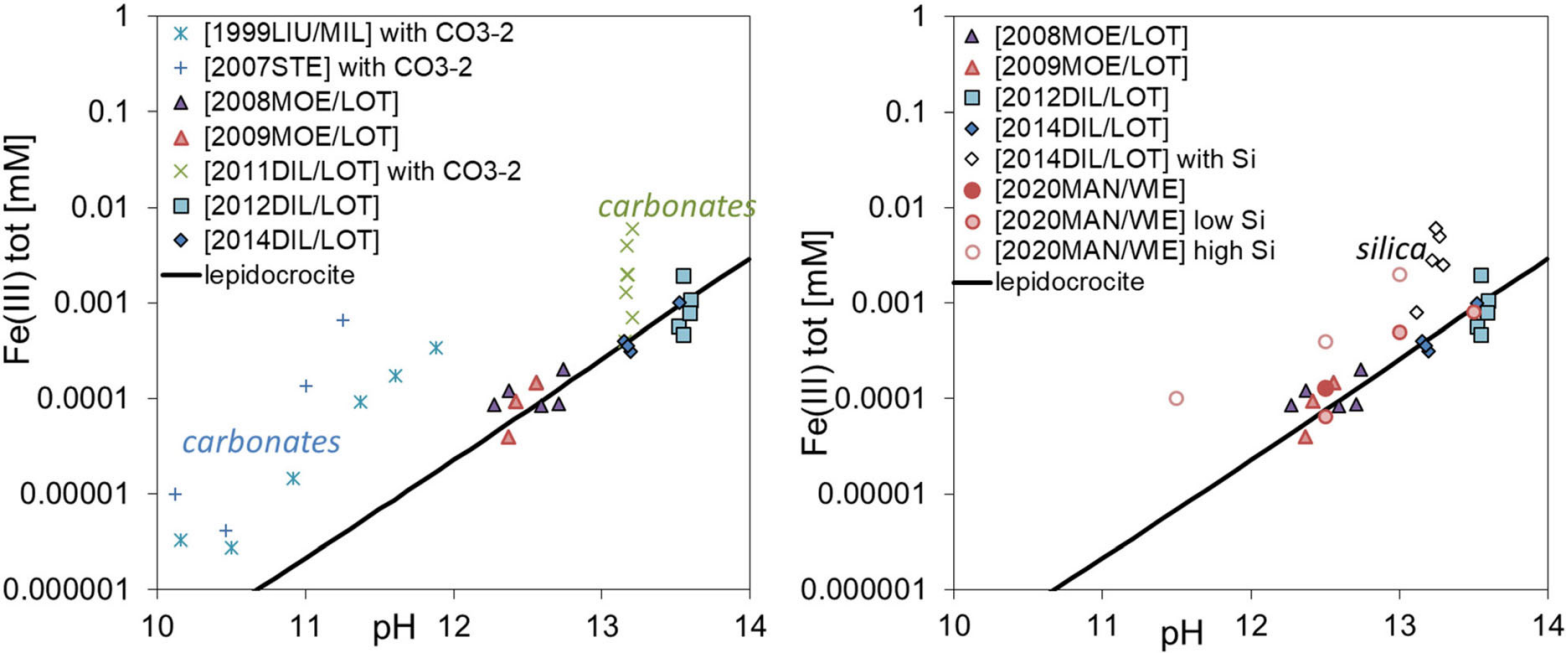
Measured solubility of iron hydroxide under alkaline conditions in the presence of carbonate (left) and silica (right); experimental data collected from [64, 87, 89, 91, 92, 123, 125, 126]. The solubility of lepidocrocite (γ-FeOOH) at 20 °C (solid lines in both figures) was calculated using thermodynamic data from Hummel and Thoenen [94]

A recent literature study by Furcas et al. [59] has highlighted that the aqueous Fe(III) concentration in the presence of iron hydroxide at high pH is increased in the presence of carbonate [87, 125, 126] and silicate [64, 123] by more than a factor of 10 (Fig. 4). This increase of the total concentrations of aqueous Fe(III) in the presence of silicate and carbonate points towards the formation of presently unidentified aqueous iron(III)-hydroxide-carbonate and iron(III)-hydroxide-silicate complexes at high pH values, which have large stability constants and strongly increase the total iron concentration in solution. The data in Fig. 4 show that the increase of the Fe(III) concentration is more pronounced at high Si concentrations (low C/S ratio of C–S–H) than at low Si concentrations (high C/S ratio of C–S–H) [123].

**Fig. 5:**
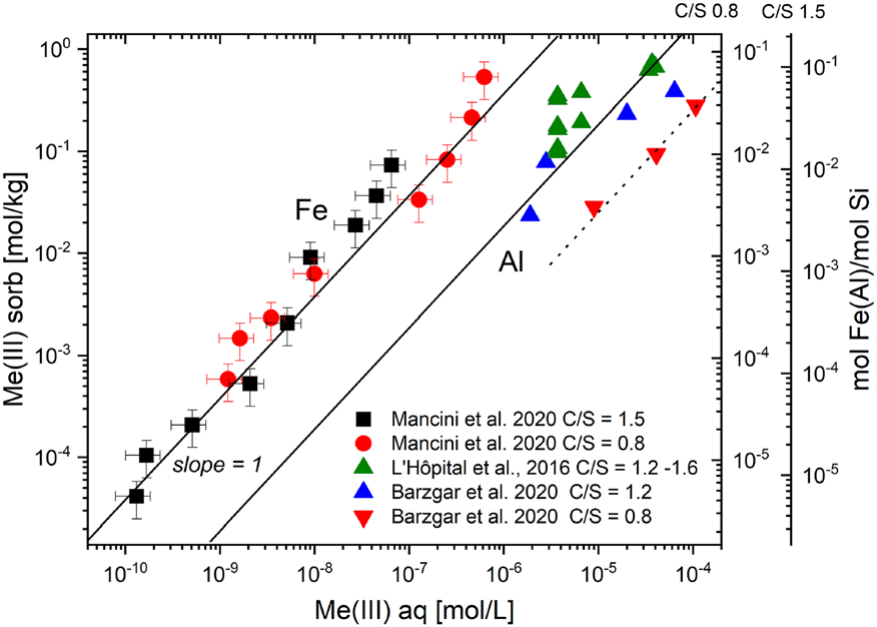
Comparison of Fe(III) and Al(III) sorption isotherms on C–S–H phases with varying C/S ratios. The left y-axis relates to the sorbed Fe(III) concentration. The molar Fe/Si or Al/Si ratios of the C–S–H phases are shown on the right y-axes. The pH in the experiments was as follows: 12.5 for Fe(III) [123], 12.4–12.6 [114] and 12.47—12.96 [112] for Al(III) (modified from Mancini et al. [119])

At high C/S ratio, the interlayer space of C–S–H is occupied by Ca^2+^ ions, water molecules and possibly alkalis, while at low C/S ratios, an aqueous electrolyte containing “interlayer” space exists [127–129]. Thus, in case of a Ca(II)-Fe(III) replacement by a cation exchange process as suggested previously [99, 122], Fe(III) uptake by C–S–H phases should be lower at high Ca concentration and high C/S ratio as previously observed for the exchange of Ca^2+^ by Sr^2+^, Ra^2+^, Ba^2+^, K^+^, and Na^+^ on C–S–H phases [130–136]. Note that the aqueous Ca concentration is almost two orders of magnitude higher in equilibrium with a C–S–H phase with C/S ratio = 1.5 as compared to a C–S–H phase with C/S ratio = 0.8 [135, 136]. However, the Fe(III) sorption isotherms in Fig. 3 clearly reveal that Ca^2+^ has no significant effect on Fe(III) uptake, which may be related to the fact that Fe(OH)_4_^−^ (and not of Fe^3+^) predominates at pH values above pH 8 (Fig. 2), thus making a cation exchange unlikely.

In strongly alkaline solution, aqueous Fe(III) is known to have a tetrahedral coordination geometry as [Fe(OH)_4_]^−^ [137]. However, at the same conditions in complex salts, Fe(III) was found to be bound preferentially in octahedral coordination [137]. The pre-edge features observed by Mancini et al. [123] in X-ray absorption near edge structure (XANES) spectra of Fe(III)-loaded C–S–H phases show that Fe(III) is bound in an octahedral coordination environment to C–S–H phases, thus confirming the Mössbauer observations [99, 122]. ^29^Si NMR relaxation data show the presence of a small amount of octahedral Fe in the silica chain at high C/S C–S–H with a maximum Fe/Si of 0.001 to 0.01 [123, 124]. The structural parameters deduced from EXAFS spectroscopy support this finding [123]. Fe(III) is surrounded by around ~ 6 O in the first coordination shell in line with octahedral coordination, and ~ 4 Si and ~ 2 Ca atoms in the second coordination shell, and about 15 O atoms in the third coordination shell. Atomic distances and numbers of neighbouring atoms suggest that Fe(III) is octahedrally coordinated to Si tetrahedra of the “dreierketten” silica chains as well as to two Ca in the interlayer space of C–S–H [123].

For C–S–H phase with a low C/S ratio of 0.8, the ^29^Si NMR data indicated that Fe(III) is not part of the silica chain, which was further supported by EXAFS spectroscopy [123]. Fe(III) is considered to form a separate secondary Ca–Si-rich phase (or clusters) on the surface of C–S–H phases based on the EXAFS data [123]. About four neighbouring Si atoms were determined at a distance of R_Fe–Si_ of ~ 3.15 ± 0.07 Å, three Ca atoms at R_Fe–Ca_ of ~ 3.19 ± 0.05 Å and two Fe(III) atoms located at a distance of R_Fe–Fe_ ~ 3.34 ± 0.05 Å.

Figure 2 shows a comparison of the Fe(III) sorption data on C–S–H phases reported by Mancini et al. [123] with those for Al reported earlier [113, 114]. The Fe(III) loading is about an order of a magnitude higher than that of Al at the same aqueous concentration. Hence, Al uptake by C–S–H phases with C/S ≥ 1 is significantly weaker as compared to Fe(III). It is noteworthy that the difference in sorption intensity is also reflected by the difference in solubility of the Al- and Fe-hydroxides in the studied pH range of 11.5–13.5 (Fig. 2). The one order higher *K*_d_ value of Fe(III) corresponds to the almost one order lower solubility of iron hydroxide. The total sorption capacity for Al on C–S–H phases, however, is higher than for Fe(III) due to the higher solubility limit of aluminium compared to iron hydroxide.

As previously discussed, Al(III) may occupy several crystallographic positions in C-A-S–H phases [113, 119, 120, 138]. At low C/S ratio, Al in tetrahedral coordination replaces Si at the bridging position of the “dreierketten” silica chains, which is not observed in the case of Fe(III). At high C/S ratio, however, Al may also be bound in octahedral coordination similar to Fe(III) in the bridging position of the silica chains stabilised by the presence of Ca^2+^ ions [119, 120]. Nevertheless, the effects of pH and Ca on Al(III) and Fe(III) uptake by C–S–H phases were found to be different [111, 112, 123], which suggests that Fe(III) is subject to a different binding mechanism than Al(III).

### Uptake of Fe(II) by cement hydrates

While Fe(OH)_2_ and Fe_3_O_4_ are formed under anaerobic corrosion, little information is available on the interaction ferrous iron (Fe(II)) with cement phases. The formation of FeS was reported in the presence of reduced sulphur species (HS^−^) [5, 6]. Hydrotalcite, a Mg–Al LDH, forms as a minor phase in cement paste and can accommodate Fe(III) replacing Al(III) in its main layer [61, 62, 106, 107, 139, 140]. Under reducing conditions, Fe(II) can partially or completely replace magnesium in Mg–Al-LDH [60, 141, 142]. Such Fe(II)-containing Mg–Al-LDH phases have also recently been observed in cements containing slags with high Fe(0) and Mg content [63], indicating that LDH phases could act as a sink for Fe(II) in magnesium-rich cements. The Fe(II) phases are more soluble when compared to the Fe(III) phases (Fig. 2c). A difference between the two systems is the minimum solubility at around pH 8 for the Fe(III) phases and at around pH 11 for the Fe(II) phases, respectively. Figure 2c shows that the Fe(II)-Al-LDH phase is less soluble at lower pH values, while at pH > 11 Fe(OH)_2_(s) is predicted to be the most stable phase. There is a potential uncertainty of at least one order of magnitude in the predicted stabilities for the LDH phases due to variability in experimental data arising from differences in sample preparation, composition, crystallinity [60]. Upon corrosion, Fe(OH)_2_ and the LDH phases are predicted to be metastable and will possibly recrystallise to magnetite with time, but in the presence of ligands like Cl^−^ in the pore solution GR might be stabilised at the expense of magnetite. The stability of the GR phase is highly dependent on the Cl^−^ concentration but also on the pH and pe [59]. With increasing pH, magnetite is favoured and higher concentrations of Cl^−^ are needed to form GR. Scoping experiments by Mancini et al. [5] also indicate the possibility of a weak uptake of Fe(II) by AFm and ettringite phases as well as in C–S–H. To the authors’ knowledge, no further studies on Fe(II) interaction with cement phases have been published to date. The reason might be that studies on Fe(II) interaction with cement phases are particularly challenging due to the limited stability field of Fe(II) in strongly alkaline conditions [143].

Mancini et al. [144] concluded from co-precipitation experiments with Fe(II) in C–S–H phases that partial replacement of Ca(II) by Fe(II) may occur in C–S–H phases, while complete stoichiometric substitution was not supported by the experimental data. Apparently, the large difference in the ionic radii of Ca(II) (*r* = 1.08 to 1.20 Å in VI-VIII coordination) and Fe(II) (*r* = 0.69 and 0.86 Å in VI low and high spin coordination, respectively) limits replacement of the two bivalent metal cations. Nevertheless, about 2% Ca (on a mol basis) was replaced by Fe(II) as determined from linear combination fitting of the XANES data, resulting in low Fe/Si ratios of ~ 0.02 in C–S–H 0.8 and ~ 0.05 in C–S–H 1.5. XRD studies reveal that the uptake of Fe increases the basal spacing for both C–S–H phases, similar to the effect of Al uptake in C–S–H, which also increases the basal spacing [114, 145]. Thus, the evidence is that an increase in the basal spacing, even at low Fe/Si ratio, could be due to Fe(II) uptake into the interlayer of C–S–H phases as previously reported for Al(III).

**Fig. 6:**
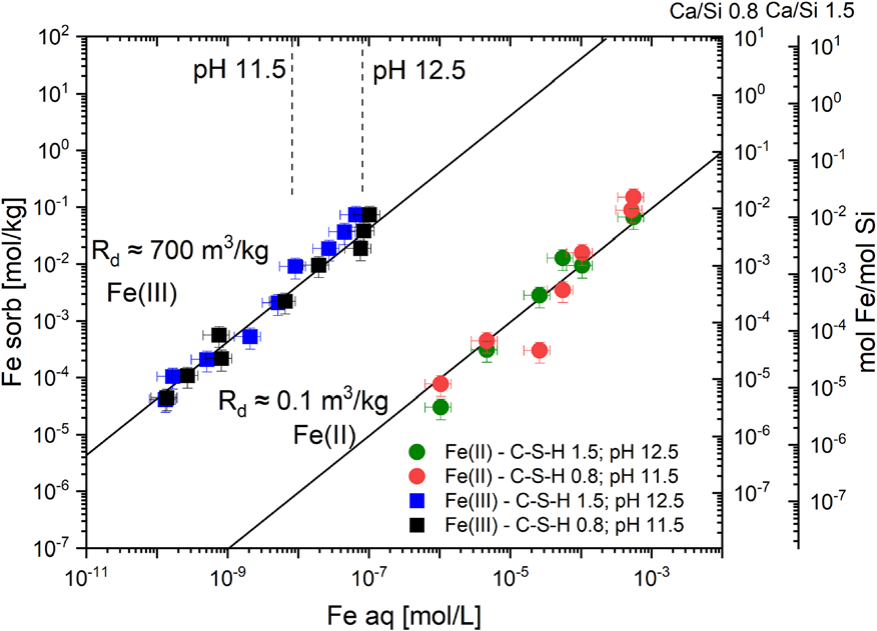
Sorption isotherms of Fe(II) on C–S–H phases with C/S ratios = 0.8 and 1.5 in comparison to those of Fe(III) on the same phases [144]. The left y-axis relates to the sorbed Fe(II) concentration. The molar Fe(II)/Si ratios of the C–S–H phases are shown on the right y-axes. The vertical dashed lines indicate the calculated solubility of Fe(OH)_2_ (adapted from Mancini et al. [144])

EXAFS spectroscopy studies on Fe(II)-loaded C–S–H phases show the absence of solid Fe(OH)_2_(s) and linearity of the sorption isotherm of Fe(II) on the C–S–H phases up to 0.5 mM Fe(II), i.e. several log units above the expected solubility of Fe(OH)_2_ of 0.9–2·10^–5^ mM at pH 11.5–12.5 (Figs. 2 and 6) [144]. This could be either due to the formation of unknown aqueous Fe-Ca–Si-hydroxide complexes or due to inhibition of Fe(OH)_2_(s) precipitation [59]. The isotherm measurements further reveal that Fe(II) sorption does not depend significantly on pH or the C/S ratio of the C–S–H phases, respectively, in the pH range between 11.5 and 12.5 as the sorption data agree within the experimental uncertainty, although a reduction in Fe(II) uptake at higher pH is expected due to the increasing concentration of aqueous Fe(OH)_3_^−^. The uptake of Fe(II) by C–S–H phases was found to be much weaker compared to Fe(III) on the same C–S–H phases, resulting in a difference between the sorption value (*K*_d_) of Fe(II) and Fe(III) of more than 3 orders of magnitude (Fig. 6).

Mancini et al. [144] further observed Fe(II) loadings up to 0.1 mol kg^−1^ on C–S–H phases irrespective of their C/S ratio and assumed the presence of equal proportions of surface-bound and interlayer-bound Fe(II) species on the basis of the EXAFS and XRD data. An alternative structure model was also discussed based on Fe(II) incorporation into the near-surface structure of C–S–H phases, which would explain the large number of neighbouring atoms around Fe(II) (i.e. total of ~ 6 (Ca + Si) neighbours) that had been derived from EXAFS spectroscopy. In summary, Mancini et al. [144] provide a plausible interpretation by assuming that (i) Fe(II) is bonded both on the surface (formation of surface complexes) and in the interlayer at about equal quantities, (ii) both sites, i.e. surface and interlayer sites, have similar affinities for Fe(II).

### Comparison between hydrolysis and sorption on C–S–H phases

The orders of magnitude difference in the sorption values of Fe(II) and Fe(III) can tentatively be explained in terms of their different hydrolysis behaviour. In the case of hydrolysis, the O–H bonds between the metal cation and water molecules of its hydration sphere are subject to pH-dependent ionisation giving rise to the formation of hydrolysed species [146]

1 $${\text{M}}\left( {{\text{OH}}_{{2}} } \right)_{n}^{z + } = {\text{ M}}\left( {{\text{OH}}} \right)\left( {{\text{OH}}_{{2}} } \right)_{n - 1}^{{\left( {z - 1} \right) + }} + {\text{ H}}^{ + }$$

Thus, the initial step of hydrolysis of a cation is the formation of the first hydrolysis product, MOH^(z−1)+^, and can generally be represented as follows:

2 $${\text{M}}^{z + } + {\text{ H}}_{{2}} {\text{O }} = {\text{ MOH}}^{{\left( {z - 1} \right) + }} + {\text{ H}}^{ + } {\text{or M}}^{z + } + {\text{ OH}}^{ - } = {\text{ MOH}}^{{\left( {z - 1} \right) + }}$$

**Fig. 7:**
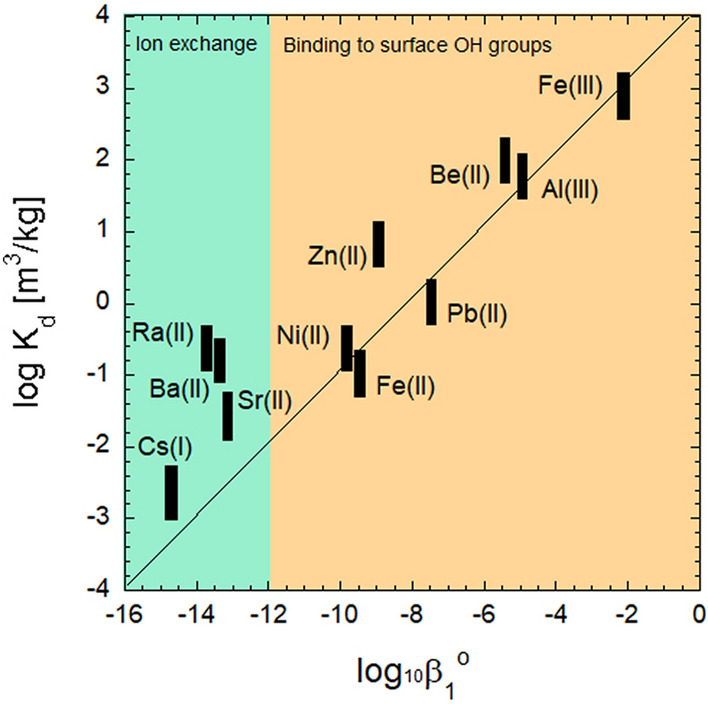
Empirical relationship between the first hydrolysis constant and the distribution coefficient (*K*_d_) of linear sorption of metal cations sorbed onto C–S–H phases (C/S ratios 1.0–1.65) in the pH range 12–12.6

The tendency of a cation to hydrolyse depends on its charge and size. For example, strongly hydrolysing cations are those that are either small (e.g. Be^2+^) and/or highly charged (e.g. Fe^3+^, Al^3+^) (Fig. 7). The tendency of metal cations to hydrolyse can also be interpreted as a tendency to coordinate OH^−^. A simplified view of the ligand exchange process at surface sites allows us to relate the bonding of aqueous OH^−^ to the metal cations to the tendency of the metal cation to coordinate to surface functional OH groups of C–S–H phases (Fig. 7). In a general way, the coordination of strongly hydrolysed metal cations (pH 12–12.5) by C–S–H phases can be described as:

3 $$\equiv {\text{S}} - {\text{O}}^{ - } + {\text{ M}}\left( {{\text{OH}}} \right)_{n}^{z - } = \, \equiv {\text{S}} - {\text{OM}}\left( {{\text{OH}}} \right)_{n - 1}^{z - } + {\text{ OH}}^{ - }$$

≡S–O^−^ represent either deprotonated OH groups bound to Ca or Si atoms on the surface or in the interlayer of C–S–H. Figure 7 shows the empirical relationship between the thermodynamic constant of the first hydrolysis reaction (log_10_β_1_°) and the distribution coefficient (*K*_d_) of linear sorption published for metal cations sorbed onto C–S–H phases (C/S ratios 1.0–1.65) in the pH range 12–12.6. Note that the comparison does not take into account the different degrees of hydrolysis of the metal cations. For example, Me(OH)_3_^−^ species dominate in the case of Fe(II), Ni(II), Pb(II), and Me(OH)_4_^−^ species dominate in the case of Fe(III) and Al(III), while for Zn(II) and Be(II), in addition to Me(OH)_3_^−^, also Me(OH)_4_^2−^ is present. However, in the case of Zn(II) and Be(II), the contribution of Me(OH)_4_^2−^ to the distribution of the hydrolytic species at pH ~ 12 is 50% at best, which suggests that the effect of differences in speciation is presumably covered within the uncertainty range of the plotted *K*_d_ values.

The hydrolysis constants have been reported by Baes and Mesmer [146], Brown and Ekberg [147] and Hummel and Thoenen [94]. The sorption data on C–S–H phases have been reported by L’Hôpital et al. [113, 148] for Al(III), Missana et al. [132] for Ba(II), Cevirim-Papaioannou et al. [149] for Be(II), Ochs et al. [150] and Missana et al. [151] for Cs(I), Mancini et al. [144] for Fe(II), Mancini et al. [123] for Fe(III), Missana et al. [152] for Ni(II), Pointeau [153] for Pb(II), Tits et al. [134] and Olmeda et al. [133] for Ra(II), Tits et al. [135] for Sr(II), and Ziegler et al. [154] for Zn(II).

It should be noted that the empirical relationship only holds for strongly hydrolysing metal cations, while it breaks down in the case of the weakly hydrolysing metal cations, such as the alkaline (log_10_β_1_° ranging from -13.84 (Li^+^) to < -14.7 (Cs^+^), [147]) and alkaline earth metals (log_10_β_1_° ranging from -12.57 (Ca^2+^) to -13.7 (Ra^2+^), [94]). For these metal cations, the interaction with the surface functional OH groups should be very weak according to the correlation shown in Fig. 7, although significant uptake by C–S–H phases has been observed in the case of alkaline and alkaline earth metals. Alkali and alkaline earth metal cations, which are positively charged even under high pH conditions, can compete with Ca^2+^ to compensate the negative surface charge of C–S–H by ion exchange [131]. Ion exchange based on charge compensation can be interpreted in terms of a non-specific binding in the diffuse layer (outer-sphere coordination) rather than by specific binding to OH groups (inner-sphere coordination). The former process is favourable because these ions form only weak M-OH bonds with aqueous hydroxyl ions.

The empirical relationship displayed in Fig. 7 suggests that in the case of Al, Be, Fe(II/III), Ni, Pb and Zn (and very likely other elements that hydrolyse already at low pH, such as the actinides and lanthanides), coordination to surface OH groups of the C–S–H phases (inner-sphere coordination) significantly contributes to the stabilisation of surface metal complexes.

### Thermodynamic data and models for modelling iron-cement interaction

Thermodynamic properties of Fe(II/III)-bearing minerals and cement phases as well as thermodynamic models for Fe(II/III) interaction with cement phases are essential for assessing the fate of iron released during iron/steel corrosion. They serve as inputs for improved modelling tools that couple geochemical reactions and transport, which could ultimately improve our understanding of the complex chemical processes at the interface between iron/steel and cement.

Over the past 15 years an increasing number of solubility data for Fe(III)-containing cement phases has been retrieved from synthesis, spectroscopy, thermogravimetric and solubility experiments, which recently have been compiled in the CEMDATA18 database [90]. CEMDATA18 contains the properties of several Fe(III)-bearing AFm and AFt phases, Al/Fe siliceous hydrogarnet, and LDH phases in addition to common cement hydrates such as C–S–H, AFm and AFt phases, hydrogarnet, and magnesium silicate hydrates (M-S–H). Solid solution models for AFm, AFt, and hydrogarnet are also included in the GEM-Selektor version of the CEMDATA18 database in order to account for Fe(III) uptake by these phases. Thermodynamic data for mixed Al–Fe-containing strätlingite, however, are missing, while strätlingite containing only Fe(III) was found to be thermodynamically unstable [88, 90]. Recent reviews of the solubility of iron (oxyhydr)oxides, sulphates and carbonates [147, 155, 156] complete the thermodynamic dataset for Fe(III). However, the strong increase in the measured Fe(III) concentration in equilibrium with iron hydroxide under alkaline conditions in the presence of silica and carbonate (Fig. 4, [59]) suggests the formation of as yet unidentified aqueous iron-hydroxide-carbonate and iron-hydroxide-silicon complexes.

In addition, preliminary models have been developed to describe the Fe(III) uptake by C–S–H based on the sorption isotherms reported by Mancini et al. [123]. The uptake of Fe(III) by C–S–H was modelled a) using a *K*_d_ value of 700 m^3^/kg for Fe(III) [5] and b) by parameterising the recently developed C–S–H solid solution model, CASH+ [131, 157] for Fe(III) on the assumption that iron can exchange at the interlayer cation sites of the tobermorite structure [21]. Fitting of the experimental data published by Mancini et al. [123] show that the model can describe very well Fe(III) uptake by C–S–H phases with different C/S ratios and at different NaOH concentrations [21]. It is intended to extend the C–S–H model to simulate Fe(III) uptake by C-A-S–H phases, i.e. Al-containing C–S–H phases, once additional experimental data on the Fe(III) uptake by C-A-S–H phases become available.

Thermodynamic data for Fe(II)-containing minerals such as magnetite, Fe(OH)_2_ and iron sulphides have been critically reviewed recently [147, 155, 156], while only a few data are available to describe the interaction of Fe(II) with cement phases. Thermodynamic data for akaganéite (β-FeO(OH,Cl)), hibbingite (Fe_2_(OH)_3_Cl), and green rust (GR(SO_4_^2−^, Cl^−^, CO_3_^2−^)) have been determined in the last years [158–160] and recently, an estimate of the solubility of Fe(II)-containing LDH (Fe(II)-Al-LDH with a formula Fe_2_Al(OH)_6_Cl) has been published [60] and was considered in our calculations. The uptake of Fe(II) by C–S–H was modelled using a *K*_d_ value of 0.1 m^3^/kg for Fe(II) by Mancini et al. [5], while no attempt to include Fe(II) in a C–S–H model has been reported yet, while it is planned to include it in the CASH + solid-solution model [131, 157]. Thermodynamic data to describe Fe(II) uptake in other cement hydrates such as ettringite, AFm or M-S–H phases are completely missing.

The compositions of the hydrated cements in contact with seawater and fresh water were thermodynamically modelled as shown in the following section using the geochemical modelling code Gibbs Energy Minimization Selektor (GEMS) [161]. General thermodynamic data were selected from the PSI/Nagra thermodynamic database [94, 162, 163] complemented with solubility products of the cement phases from the CEMDATA18 database [90] and additional Fe(II) phases as detailed above. Note that in the modelling data presented in the following section, the formation of quartz (SiO_2_), dolomite (CaMg(CO_3_)_2_), pyrite (FeS_2_), goethite (α-FeOOH), and hematite (α-Fe_2_O_3_) in the hydrated cements was suppressed in the calculations for kinetic reasons. Note further that allowing the formation of hematite and goethite would supress the formation of siliceous hydrogarnet ((CaO)_3_(Al,Fe)_2_O_3_(SiO_2_)_0.84_(H_2_O)_4.32_).

## Thermodynamic modelling of iron-cement interaction

Very few studies have modelled processes at the iron/steel-cement interface and the evolution of geochemical conditions and mineral compositions at this interface. The current situation may be a consequence of the limited thermodynamic and kinetic data available on the interaction of Fe(II) and Fe(III) with cement phases. The following examples aim at providing insight into the chemical evolution at the iron-cement interface based on the currently available thermodynamic data.

### Fate of Fe(0/II/III) in iron-rich cement systems

The availability of thermodynamic data allows the fate of Fe(III) in cementitious systems to be predicted and compared with experimental data. For example, thermodynamic modelling was applied to predict the time-dependent evolution of the Fe(III) speciation in PC [95, 96], blended cements [108, 164, 165] as well as in ferrite belite-ye'elimite cements [98]. In PC as well as in CaO rich blended cements and ferrite belite-ye'elimite cements, the formation of Fe/Al-siliceous hydrogarnet has been predicted [95, 96, 98] in agreement with experimental results [65, 95, 98]. The formation of Fe-ettringite is predicted by thermodynamic modelling only in the presence of a surplus of CaSO_4_ [96]. In CaO-poor cements, e.g. in blended cements with a high amount of SiO_2_ [7, 108] as well as in leached or carbonated surfaces [166, 167], the formation of ferrihydrite is predicted instead.

The corrosion of Fe(0) present in nano-sized particles in slag cements is relatively slow as Fe(0) has been observed after several years of reaction [3, 5]. Thermodynamic modelling of slag cements predicts reducing conditions and the formation of FeS [5, 6, 168] in agreement with the spectroscopic investigations of Mancini et al. [5] that show the presence of metallic iron along with minor proportions of iron sulphide and magnetite in alkali activated slag cements.

**Fig. 8:**
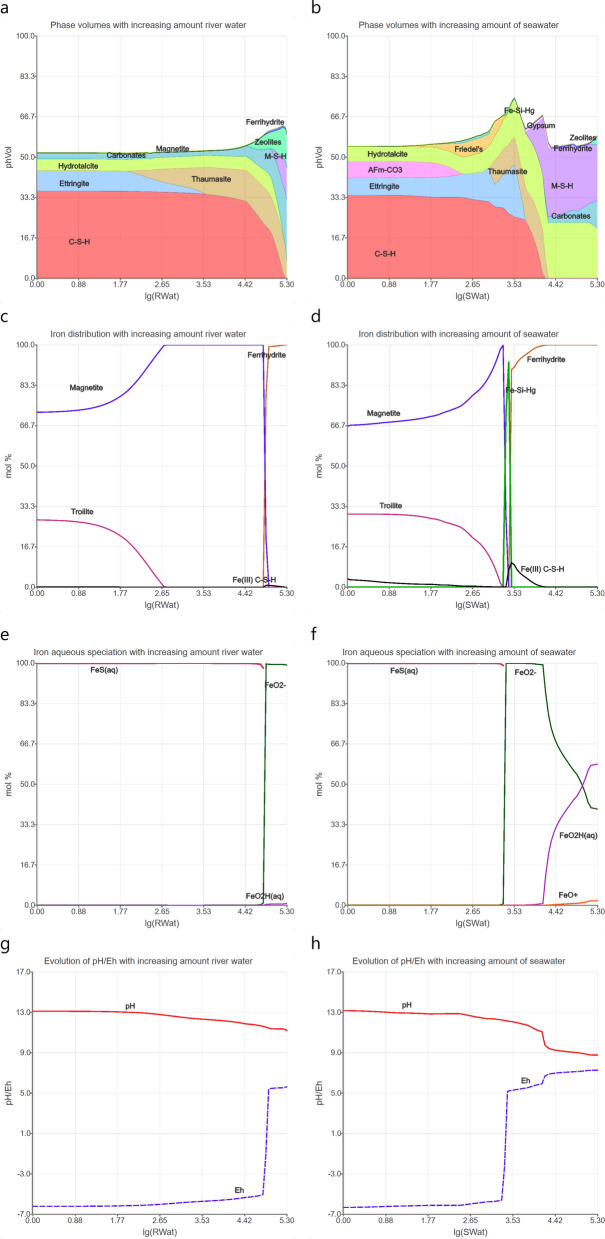
Predicted evolution of slag-containing concrete exposed to increasing amounts (in log10 scale) of river water (RWat) and North Sea seawater (SWat) based on thermodynamic modelling (modified from Mancini et al. [5]). **a**, **b)** hydrate assemblage, **c**, **d**) iron in solid phases, **e**, **f**) aqueous speciation of iron, and **g, h**) pH (solid line) and *E*_h_ (dashed line)

For slags blended PCs the formation of iron sulphide and magnetite is predicted ([5], Fig. 8) in agreement with experimental observations using standard analytical techniques in combination with synchrotron-based techniques and thermodynamic modelling [5]. Iron sulphide, iron (oxyhydr)oxides, and siliceous hydrogarnet in addition to Fe(0) have been identified in blended slag cement hydrated in contact with river water for up to 7 years. These findings suggest passivation of Fe(0) as observed on steel bars.

In contrast, no Fe(0) was observed in slag cements exposed to seawater, which indicates accelerated oxidation of Fe(0) by Cl^−^. Iron sulphide, iron(II) hydroxide and oxide, iron(III) (oxyhydr)oxide and siliceous hydrogarnet have been identified as corrosion products under these conditions [5].

The interaction of the Fe(0)-containing slag cements with river and seawater was thermodynamically modelled as displayed in Fig. 8. Figure 8a/b show on the left the initial compositions of the hydrated cements, while on the right the gradual changes towards the surface of the concretes exposed to either river or seawater are displayed. The vicinity of the concretes to the river or seawater was simulated by increasing the amount of exposure solutions [166, 169].

Figure 8a/c/e depict the modelling for the slag-containing concrete exposed to river water. This is an update of the modelling previously reported by Mancini et al. [5] with the extension that the CASH + model [131, 157] was used, including a preliminary extension accounting for Fe(III) uptake as well as Fe(II)-containing LDH (Fe_2_Al(OH)_6_Cl). C–S–H phases, ettringite, strätlingite, monocarboaluminate, hydrotalcite, and calcite are predicted to exist in the non-exposed zone at an initial pH of 13.7 (left side of Fig. 8a/c/e). Close to the surface, the formation of calcite occurs due to the ingress of carbonate present in the river water and of thaumasite (Ca_3_(SiO_3_)(CO_3_)(SO_4_)·15H_2_O) towards the inner part, where the pH drops to 12. On the surface of the concrete, indicating more intense exposure to river water, the formation of M–S–H phases and zeolites is predicted, while C–S–H is destabilised and the pH value drops to 11. Magnetite (Fe_3_O_4_) and troilite (FeS) are predicted as the iron minerals that are thermodynamically stable in the non-exposed slag blended cement paste (left part in Fig. 8c), indicating conversion of Fe(0) into these minerals with time. The redox potential (*E*_h_) of − 650 mV indicates strongly reducing conditions in the unaltered part (Fig. 8e). The formation of Fe(II)-containing LDH (Fe_2_Al(OH)_6_Cl) is not predicted. The formation of Fe(OH)_3_ instead of Fe_3_O_4_ and FeS is predicted at the surface of concrete, i.e. in areas more intensely exposed to river water. This coincides with an increase in *E*_h_ towards more oxidising conditions. Very low Fe concentrations of 0.01 µmol/L at maximum are predicted, resulting in very low Fe contents of the C–S–H phases (molar Fe(III)/Si ratio ≤ 0.001; molar Fe(II)/Si  ratio < 1 × 10^–9^) based on the sorption data reported by Mancini et al. [123].

Figure 8 also shows the modelling results for the slag-containing concrete exposed to seawater. Again, this figure is an update of the modelling reported previously by Mancini et al. [5], extended to include the CASH + model. In the non-exposed region of this concrete (left side of Fig. 8b/d/f), the initial composition of the hydrate assemblage is predicted to be composed of C–S–H, monocarboaluminate, hydrotalcite, and ettringite at an equilibrium pH of 13.9. Increasing exposure to seawater results in the formation of Friedel's salt (Ca_4_Al_2_Cl_2_(OH)_12_·4H_2_O)). The pH strongly decreases (Fig. 8f) upon complete dissolution of C–S–H, indicated also by the dissolution of monocarboaluminate. The formation of ettringite, thaumasite, and gypsum occurs closer to the surface due to the presence of sulphate, carbonate, and magnesium in seawater. Friedel’s salt, C–S–H phases and ettringite decompose at the surface, leading to the formation of M-S–H phases, carbonates, and hydrotalcite in agreement with the changes observed experimentally [9, 166]. The pH is ~ 9 close to the surface of concrete, which is controlled by the presence of calcite. In the hydrated, non-exposed blended cements, iron sulphides and magnetite are predicted as the thermodynamically most stable Fe-containing phases, indicating conversion of Fe(0) into these minerals with time. Fe/Al siliceous hydrogarnet seems to be stable only in a small range of seawater addition, while no Fe(II)-containing LDH is predicted. At the highest exposure to seawater, that is on the surface of the concrete where oxidising conditions dominate, ferrihydrite is the only Fe-rich phase predicted to be stable with a small amount of Fe(III) being taken up by C–S–H phases in the stability range of C–S–H. Again, the amount of Fe(II) taken up by C–S–H phases is negligibly small. The evolution of the redox conditions in the concrete exposed to seawater is comparable to that in the concrete exposed to river water. A negative *E*_h_ value is given for the pristine paste, while a strong increase occurs after complete dissolution of Fe_3_O_4_ and FeS.

Negative *E*_h_ values were also found in a hydrated CEM-V/A cement, which is a ternary blended cement consisting of 50 wt.% Portland cement, 25 wt.% blast furnace slag and 25 wt.% fly ash, based on the presence of reduced Fe phases and (bi)sulphides [170]. It was found that the equilibrium between half-reactions of Fe(OH)_2_/Fe(OH)_3_ couple and S^0^/S^2−^ couple resulted in an *E*_h_ value of − 450 mV at pH 13.5 [170]. This redox potential matched well with the experimental *E*_h_ value imposed by steel and the observed corrosion products in contact with the synthetic cement pore solution (pH ~ 13.5). By applying the Nernst equation on the different corrosion product couples (Fe^0^/Fe(OH)_2_, magnetite/hematite, or magnetite/goethite couple) and the redox-sensitive molecular probe (U(VI), Se(IV), Mo(VI), or Sb(V) anion) [171], it was shown that the *E*_h_ value of − 450 mV is probably controlled by the amorphous Fe(OH)_2_/Fe(OH)_3_ or (Fe_1−x_,Ca_x_)(OH)_2_/Fe(OH)_3_ couple forming on the surface layer of bulk Fe(II/III) (oxyhydr)oxides. The *E*_h_ value derived from bulk magnetite/goethite couple was computed to be lower, − 576 mV at pH ~ 13.5 [171], which is comparable to the calculated *E*_h_ values (− 650 mV at pH 13.7) in Figs. 8e/f. The good match again confirms that the negative *E*_h_ values are mainly controlled by Fe(II/III) (oxyhydr)oxides and FeS, regardless of their origins from steel corrosion products or supplementary cementitious materials.

The thermodynamic modelling suggests that, under the given conditions and for cement compositions at thermodynamic equilibrium, Fe(III) is mainly associated with magnetite, while the extent of Fe(III) uptake by C–S–H phases is small, although Fe(III) interaction with C–S–H phases is strong and the amount of C–S–H in the concretes is large. Fe(II) is mainly associated with Fe_3_O_4_ and FeS, and the amount of Fe(II) accommodated by C–S–H is very low. Under reducing conditions (equilibrium with FeS and magnetite), the iron in solution is predicted to form a neutral complex with sulphur, i.e. FeS(aq), which dominates the ferrous iron speciation in solution. Under oxidising conditions, the iron hydrolysis species (Fe(OH)_4_^−^ or FeO_2_^−^ and Fe(OH)_3_(aq) or FeHO_2_(aq)) are the dominant chemical forms of ferric iron.

The above findings suggest a pronounced effect of the Fe(II/III) speciation on the iron mobility and support the idea that aqueous iron species originating from corrosion can be strongly retarded by the cement paste and thus will be retained close to the iron/steel-concrete interface. However, competition between iron and other cations in solution for incorporation into C–S–H might occur [123, 144].

### Reactive transport and mechanical modelling of iron-cement interaction 

Reactive transport modelling allows the transport and chemical reactions of multiple solutes (and gases) and their chemical interaction with materials as a function of time and space to be predicted. Reactive transport modelling is widely used in earth sciences and in particular in conjunction with assessing the long-term performance of DGRs for radioactive waste (e.g. [172, 173]. It has become an extremely valuable tool for predicting the evolution of materials at interfaces with large chemical gradients, such as between cementitious and clayey materials (e.g. [174–177] and at iron-clay interfaces (e.g. [19, 22, 24–26, 178]. Nevertheless, very few studies have reported the development of reactive transport models, which allow iron corrosion and the evolution of corrosion fronts in cementitious materials to be modelled in terms of coupled transport and chemical processes. For example, De Windt et al. [20] used the reactive transport code HYTEC to simulate steel corrosion in the Toarcian argillite in oxic conditions in connection with modelling oxygen gas diffusion in a disposal cell of radioactive waste. Odorowski et al. [23] published a modelling study on the effect of metallic iron on the oxidative dissolution of uranium oxide, thus accounting for iron corrosion in anoxic conditions in Callovo-Oxfordian claystone. In both studies metallic iron, goethite, magnetite, melanterite, pyrite and siderite were considered as the only possible Fe-containing solid phases, while ferrous and ferric iron-containing cement phases were not considered in the thermodynamic set-up of the modelling approaches. Reactive transport models involving iron corrosion and the distribution of corrosion products in the cementitious materials have recently been proposed [16–18, 21]. These models account for iron dissolution, ferrous ion diffusion, oxidation and precipitation of ferrous and ferric iron-containing compounds. A sensitivity analysis of the different processes revealed that the oxidation rate of ferrous to ferric iron and the matrix factors (constrictivity, tortuosity) influencing the transport of the ferrous ion are the most important parameters that can determine the distribution of corrosion products at the iron-cement interface [16]. The development of reactive transport models requires a solid understanding of the interaction of ferrous and ferric iron with cement phases and well-established thermodynamic data for the ferrous and ferric iron-containing minerals that form in cementitious environments.

Mechanical modelling of iron/steel corrosion-induced cracking in concrete have been reported since several decades ago [179–183]. Many such models needed a list of physical properties of the corrosion product as input, such as the density, volume expansion rate, elastic modulus, creep coefficient, etc. The rate of the corrosion product formation is also often a parameter to be either fitted or presumed. Despite the highly variable (and so far not completely clear) speciation of iron at the iron-cement interface, most mechanical models seem to be able to only adopt a single phase of the hydration products throughout the corrosion process. Due to such a simplification and uncertainty of material parameters, complicated numerical/analytical modelling often does not lead to more accurate predictions than those obtained via simple, empirical models [182]. A coupled chemical-transport-mechanical modelling may provide more realistic prediction to rebar corrosion induced degradation. So far, work of this kind is very rare.

## Conclusions

The interaction of Fe(II) and Fe(III) released in the course of iron/steel corrosion with adjacent matrices, such as glass, cement paste, etc. is a phenomenon commonly observed. This process may have a feedback on the corrosion process such as acceleration or inhibition/passivation, and further, may change the composition of the matrices at the interface with iron/steel. The formation of corrosion products at the steel-concrete interface is well known. Recent experimental studies indicate that ferrous and ferric iron generated during the course of Fe(0) corrosion may also interact with cement phases present at the interface between steel and concrete.

Fe(III) can potentially replace Al(III) in Al-containing cement phases such as AFm, AFt phases and Fe/Al siliceous hydrogarnet, which is often modelled in terms of solid solutions. Fe/Al siliceous hydrogarnet was found to be the thermodynamically most stable Fe(III)-containing phase in PC paste and it was recently identified as corrosion product formed at the iron-cement interface. At the time being, however, the impact of the formation of the Al/Fe siliceous hydrogarnet on the corrosion process has not yet been entirely clarified, i.e. whether its formation contributes to iron passivation or it may even accelerate iron corrosion. In addition to the aforementioned siliceous hydrogarnet, also C–S–H phases were found to accommodate Fe(III), predominately in the interlayer, as previously observed for Al(III). In general, it has been observed that Fe(III)-bearing cement phases and Fe(III) (oxyhydr)oxides have a very low solubility, which results in very low Fe(III) concentrations in solution. The very low solubility of Fe(III)-bearing cement phases and the strong uptake of Fe(III) by C–S–H phases suggests strong retention of Fe(III) close to the steel-concrete interface.

In contrast to Fe(III), interaction of Fe(II) with cement phases is significantly weaker, most likely forming surface-bound complexes on AFm and AFt phases, while partial uptake of Fe(II) into the interlayer of C–S–H phases was suggested. In any case, replacement of Ca(II) by Fe(II) is very limited in cement phases, in particular in C–S–H phases.

Under the alkaline conditions (pH > 11) prevailing in cementitious systems, the concentrations of Fe(II) and Fe (III) ions (depending on the redox conditions) are very low with most of the iron being hydrolysed in the form of Fe(II)(OH)_3_^−^ and Fe(III)(OH)_4_^−^ species, respectively. However, the strong increase of measured Fe(II) and Fe(III) concentrations equilibrated with iron hydroxide in the presence of silica and carbonate indicates the presence of as yet unidentified aqueous iron-hydroxide-carbonate and iron-hydroxide-silicon complexes, which greatly increases the total iron concentration in solution and thus the transport from the corroding steel bar into the cementitious matrix. In contrast to Fe(III), Fe(II) uptake by C–S–H is much weaker, thus facilitating the mobility of Fe(II) as compared to Fe(III).

Under anaerobic conditions, the most stable corrosion product in highly alkaline conditions (pH > 11) is magnetite, while goethite is thermodynamically stable in oxic conditions. The formation of the stable iron oxides/hydroxides can be inhibited, depending on kinetics, temperature and local composition, resulting in the formation of different intermediate metastable phases. The presence of different ligands, such as chloride, sulphate or carbonate, can lead to the formation of complexes with iron, depending on their aqueous concentrations. For example, iron chloride species play an important role in the formation of Cl^−^-containing green rust GR1(Cl^−^) and may enhance the solubility and transport of iron.

Thermodynamic modelling of the long-term fate of Fe(0) in slag cements exposed to fresh and seawater reveal that Fe_3_O_4_ and FeS are the most stable Fe-containing products resulting from Fe(0) corrosion under anaerobic conditions. The proportion of Fe(II/III) taken up by C–S–H phases is much lower compared to Fe associated with the iron minerals. In general, it has been observed that Fe(III)-bearing cement phases and Fe oxides/hydroxides have a low solubility, which results in low Fe(III) concentrations in solution. The thermodynamic modelling of the long-term fate of Fe(0) in slag cements indicates that the formation of iron-containing minerals, such as magnetite and pyrite under anaerobic conditions, are the main sinks for iron upon release in the course of iron/steel corrosion rather than Fe(II) and Fe(III) uptake by cement phases. Nevertheless, further studies are needed to support the current, preliminary assessment, in particular to explore the possibility of ferrous and ferric iron interactions with cement phases at the iron/steel-cement interface under various geochemical conditions.

Very few studies have reported the development of reactive transport models that allow iron/steel corrosion at the iron/steel-concrete interface to be modelled by coupling transport and chemical processes. The modelling approach requires the availability of thermodynamic descriptions of the interaction of ferrous and ferric iron with cement phases (sorption, precipitation of Fe-containing cement phases). Such modelling can provide new insights with regard to the distribution of corrosion products at the iron/steel-concrete interface and their consequence for the durability of construction materials as well as the impact of steel corrosion on the engineered barriers in DGRs for radioactive waste.
